# Microbiome and Biocatalytic Bacteria in Monkey Cup (*Nepenthes* Pitcher) Digestive Fluid

**DOI:** 10.1038/srep20016

**Published:** 2016-01-28

**Authors:** Xin-Yue Chan, Kar-Wai Hong, Wai-Fong Yin, Kok-Gan Chan

**Affiliations:** 1 Division of Genetics and Molecular Biology, Institute of Biological Sciences, Faculty of Science, University of Malaya, 50603 Malaysia

## Abstract

Tropical carnivorous plant, *Nepenthes*, locally known as “monkey cup”, utilises its pitcher as a passive trap to capture insects. It then secretes enzymes into the pitcher fluid to digest the insects for nutrients acquisition. However, little is known about the microbiota and their activity in its pitcher fluid. Eighteen bacteria phyla were detected from the metagenome study in the *Nepenthes* pitcher fluid. *Proteobacteria*, *Bacteroidetes* and *Actinobacteria* are the dominant phyla in the *Nepenthes* pitcher fluid. We also performed culturomics approach by isolating 18 bacteria from the *Nepenthes* pitcher fluid. Most of the bacterial isolates possess chitinolytic, proteolytic, amylolytic, and cellulolytic and xylanolytic activities. Fifteen putative chitinase genes were identified from the whole genome analysis on the genomes of the 18 bacteria isolated from *Nepenthes* pitcher fluid and expressed for chitinase assay. Of these, six clones possessed chitinase activity. In conclusion, our metagenome result shows that the *Nepenthes* pitcher fluid contains vast bacterial diversity and the culturomic studies confirmed the presence of biocatalytic bacteria within the *Nepenthes* pitcher juice which may act in symbiosis for the turn over of insects trapped in the *Nepenthes* pitcher fluid.

Tropical carnivorous plant, *Nepenthes* (locally known as monkey cup) has been recognized to have evolutionarily modified leaves as the external digestive organs via leaf epiascidiation process in order to acquire nutrients from preys trapped within the pitcher cups^1^. During the digestive process, *Nepenthes* reduces the pH of its fluid to facilitate the enzymatic reactions and to regulate the bacterial population present in the micro-habitat^2^,^3^. Moreover, *Nepenthes* secretes antimicrobial and antifungal compounds such as thaumatin-like protein, plumbagin, 7methyl juglone and naphthoquinones^2^,^4^,^5^. These compounds have been reported to play an important role in *Nepenthes plant* self-defense mechanism against pathogens as well as creating an unfavorable environment for the growth of microorganisms^2^,^4^,^5^. Despite these findings, the true representation of native microbial diversity in the presence of selective force created by the plant is yet to be defined. In this study, we investigated the microbiome of the *Nepenthes* digestive fluid using metagenomic approach in order to illustrate an overview of microbiota in this unique habitat.

In general, the *Nepenthes* pitcher is believed to act as a passive insect trap and produces several groups of enzyme in order to breakdown the insect thus acquiring nutrient upon enzymatic digestion^5^,^6^,^7^. The roles of *Nepenthes* pitcher fluid microbiome has not been studied extensively. To address the gap of knowledge, we conducted the biocatalytic activities study on the bacterial isolated from *Nepenthes* juice. The aim of this study is to investigate selected biocatalytic activity of the bacteria isolated from *Nepenthes* fluid. These are the main activities involved in the degradation of insect and plant debris trapped in the pitcher, churning it into nutrient for the *Nepenthes* host^8^.

## Results

### Metagenomic Studies

A total of 0.5 million reads (585,046) was generated from metagenomic sequencing. The average read length after quality trimming is 351 bp. Based on the rarefaction curve (Supplementary Figure S1), the alpha diversity of *Nepenthes* pitcher fluid sample H1 is 31.44. These metagenomic dataset was deposited in sequence read archive (SRA) with the accession number SRR916131.

### Microbiome in *Nepenthes* Digestive Fluid

**Figure 1:**
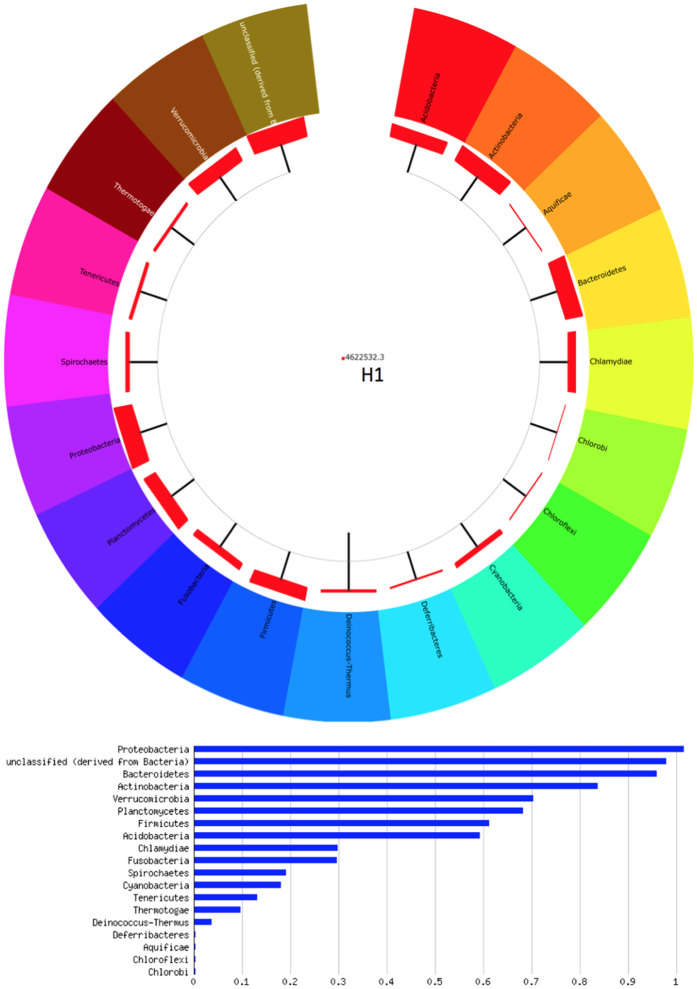
Bacteria composition in *Nepenthes* pitcher fluid H1 at phylum level. *Nepenthes* bacteria 16S rDNA sequences were compared to Ribosomal Database Project (RDP). *Proteobacteria* is the dominant phylum in the *Nepenthes* pitcher fluid H1 microbiome followed by *Bacteroidetes*, *Actinobacteria*, *Verrucomicrobia*, and *Planctomycetes*.

Metagenome result shows that *Nepenthes* pitcher fluid contained vast bacterial diversity. Eighteen phyla (Fig. 1) including 29 classes, 53 orders, 112 families and 238 genera and 616 species were identified by metagenome analysis. The dominant bacteria phyla in *Nepenthes* pitcher fluid sample H1 were *Proteobacteria* followed by *Bacteroidetes*, *Actinobacteria*, *Verrucomicrobia* and *Planctomycetes* (Fig. 1). A total of 27% of the sequencing read does not match to any DNA sequence in the RDP database. Thus, these reads were listed as “unclassified” (Fig. 1).

*Acidobacteria* which is commonly found in environment with lower pH, was not the dominant bacterial group in this slightly acidic *Nepenthes* pitcher fluid (pH6). It contributed only 0.3% of the total bacteria in *Nepenthes* fluid. A total of three members from the phylum *Acidobacteria* were identified within the *Nepenthes* fluid, namely *Terriglobis saanensisi* (98%), *Acidobacterium capsulatum* (0.9%) and Candidatus *Koribacter versatilis* (0.8%).

### Bacteria Identity

Eighteen bacteria were isolated from *Nepenthes* pitcher fluid sample H1. These bacteria were identified by MALDI-TOF MS Biotyper analysis on the bacterial protein profile and the phylogenetic analysis of the bacteria 16S rDNA gene. The bacteria identity obtained from both methods is listed in Table 1. The DNA sequences of 16S rDNA genes were deposited in GenBank with the accession numbers listed in Table 1.

### Biocatalytic Activity of the Bacterial Isolates

We conducted the amylolytic, proteolytic, cellulolytic, xylanolytic and chitinolytic screenings and the results are shown in Table 2. *Bacillus* sp. strains H1a and H1m, *P*. *aeruginosa* strain H1l, *Sphingobacterium* sp. strain H1ai, *S. marcescens* strain PH1a and H1q, *Pseudomonas* strain PH1b, *Myroides odoratimimus* strain H1bi and *Microbacterium paraoxydans* strain DH1b possessed proteolytic activity, specifically caseinase activity, as indicated by the formation of cleared zones on the skim milk agars.

The ability of the 18 isolates in the degradation of polysaccharide of different degree of complexity was tested using starch, cellulose and xylan as substrates. Among the tested isolates, *Bacillus* strains H1a, H1m and *Sphingobacterium* sp. strain H1ai shows amylolytic activity. The degradation of the iodine/starch complex by the bacteria released iodine into the medium forming yellow zone around the colony on the dark blue background.

On the other hand, the cellulose degradation by cellulolytic bacteria was identified by the Congo red discoloration which resulted in the formation of light yellow zone or clear zone around the colony on cellulose agar^9^. Yellow zone surrounding the bacterial colony on cellulose agars was observed upon inoculation of *Bacillus* sp. strains H1a and H1m, *Sphingobacterium* sp. strain H1ai, *Pseudomonas* sp. strains H1h and PH1b, *M. odoratimimus* strain H1bi, *S*. *marcescens* strain PH1a and *M. paraoxydans* strain DH1b, indicating cellulolytic activity.

The screening for xylanase activity was conducted by fluorescence-based protocol with EnzChek Ultra Xylanase Assay. The xylanase substrate was labeled with fluorescence dye as described by the manufacturer. Xylanase-producing bacteria cleaved the xylosidic linkages of the xylanase substrate and released the xylose(s) as well as the flurophore from the mentioned xylanase substrate. The fluorescence level was compared with the positive control and the fluorescence reference control. Results show that *Pseudomonas* sp. strains H1h, *P*. *aeruginosa* strain H1l, *Sphingobacterium* sp. strain H1ai, and *Pseudomonas* sp. strains PH1b exhibited xylanolytic activity.

In this study, β-*N*-acetylglucosaminidase, chitobiosidase and endochitinase activities were tested by supplying bacterial isolates with chito oligosaccharides (4-nitrophenyl *N*-acetyl-β-D-glucosaminide, 4-nitrophenyl *N*,*N*’diacetyl-β-D-chitobioside or 4-nitrophenyl β-D-*N*,*N*’,*N*”-triacetylchitotriose) with nitrophenol added to the substrate’s terminal non reducing end. The chitinase produced by the bacterial isolates hydrolysed the β(1–4) glycosidic bond of the chitinase substrate supplied released *p*-nitrophenol into the solution. The *p*-nitrophenol was ionized by the stop solution (sodium carbonate) into *p*-nitrophenylate ion which appeared as yellow color to human eye and absorbed at the absorption 405 nm.

Analysis shows that *S. marcescens* strain H1q and PH1a exhibited the highest β-*N*-acetylglucosaminidase, chitobiosidase and endochitinase activities (Supplementary Table S1). These bacteria digested the substrate 4-nitrophenyl *N*-acetyl-*β*-_D_-glucosaminide at the kinetic of releasing 2.15 nmole *p*-nitrophenol per min in 1 ml of solution, 4-nitrophenyl *N*,*N*’diacetyl-*β*-_D_-chitobioside at the releasing kinetic of 3.75 and 3.86 nmole min^−1^ ml^−1^ and 4-nitrophenyl *β*-_D_-*N*,*N*’,*N*” -triacetylchitotriose at the releasing kinetic of 2.54 and 2.06 nmole min^−1^ ml^−1^, respectively (Supplementary Table S1). However, other *Serratia* spp. isolates possessed insignificant β-*N*-acetylglucosaminidase, chitobiosidase and endochitinase activities.

On the other hand, *Klebsiella oxytoca* strain H1g and *Pseudomonas* sp. strain PH1b exhibited relatively high chitobiosidase activity by releasing *p*-nitrophenol from *N*,*N*’diacetyl-*β*-_D_-chitobioside at the 2.38 and 3.79 nmole min^−1^ ml^−1^, respectively (Supplementary Table S1). In addition, *Pseudomonas* sp. PH1b also displayed a relatively significant endochitinase activity in which it releases *p*-nitrophenol from 4-nitrophenyl *β*-_D_-*N*,*N*’,*N*”-triacetylchitotriose at the rate of 3.79 and 1.41 nmole min^−1^ ml^−1^, respectively (Supplementary Table S1).

### Bacterial Genome

Genomic study was carried out on each of the bacterial isolated from the *Nepenthes* fluid. The estimated genome size, average coverage, G + C content, number of contigs and GenBank accession number of each bacteria are listed in Table 3. The size of bacteria genome in this study range from 3.1 to 7.4 Mbps and the genome G + C content ranges from 34% to 71.3% (Table 3). This Whole Genome Shotgun project has been deposited at DDBJ/EMBL/GenBank under the accession number of AYME00000000-AYMV00000000. The version described in this paper is version AYME01000000-AYMV01000000.

### Chitinase Gene Determination

A total of 15 bioinformatically predicted chitinase-encoding genes were identified from the mentioned bacterial genomes and the sequences were deposited at DDBL/EMBL/GenBank with the accession numbers KT921876-KT921890 (Supplementary Table S2).

**Figure 2:**
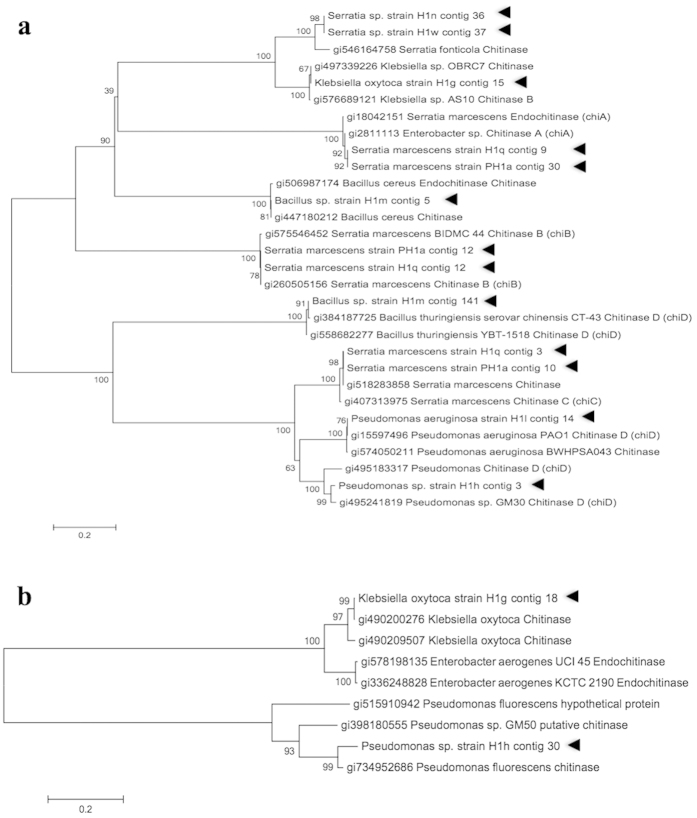
Chitinase genes from bacteria isolated from *Nepenthes* pitcher fluid H1. This phylogenetic tree was constructed by comparing the amino acid sequences of the bacteria chitinase genes against NCBI non-redundant (nr) database. The positions of the chitinase genes from bacterial isolates were indicated by solid triangles. (**a**) Thirteen chitinase genes were grouped into glycoside hydrolase (GH) family GH18 and, (**b**) 2 chitinase genes were classified into GH19.

The amino acid sequences of the predicted genes were compared against NCBI-nr database followed by categorization of sequences according to members of glycoside hydrolases (GH). Thirteen of these predicted chitinase genes were grouped under the GH 18 family chitinase (Fig. 2A) and 2 of the genes were grouped into GH 19 family chitinase (Fig. 2B). The predicted chitinase gene sequences from *S*. *marcescens* H1q and PH1a were identical. Therefore, only chitinase genes from *S*. *marcescens* H1q were selected for subsequence studies. Similarly, chitinase gene sequences of *S*. *fonticola* strain H1n and strain H1w were identical. Hence only chitinase gene from *S*. *fonticola* H1n was selected for further studies. The selections were based on alphabetical order of the strain name. These predicted chitinase genes activity were validated empirically where the predicted chitinase genes were heterologously cloned and expressed in *Escherichia coli* BL21 followed by chitinase assay on these transformants.

A total of 6 out of 11 transformants exhibited chitinolytic activity. *β*-*N*-acetylglucosaminidase cloned from contig 15 of *K*. *oxytoca* H1g and contig 36 of *S*. *fonticola* H1n showed hydrolysis of 4-nitrophenyl *N*-acetyl-*β*-_D_-glucosaminide and thus releasing *p*-nitrophenol at 1.58 and 1.45 nmole min^−1^ ml^−1^, respectively (Supplementary Table S1). The transformants which carried the chitobiosidase-encoding genes of *Pseudomonas* H1h, *P*. *aeruginosa* H1l, *Bacillus* sp. H1m, and *S. marcescens* H1q were capable to hydrolyse the *β*-1,4-glycosidic bond of *N*,*N*’diacetyl-*β*-_D_-chitobioside at 3.70, 2.47, 4.56 and 1.97 nmole min^−1^ ml^−1^, respectively (Supplementary Table S1). Lastly, the transformant which carried the chitinase genes of *Pseudomonas* H1h, *P*. *aeruginosa* H1l, *Bacillus* sp. H1m, and *S. marcescens* H1q hydrolysed the *β*-1,4-glycosidic bond of 4-nitrophenyl *β*-_D_-*N*,*N*’,*N*”-triacetylchitotriose and thus releasing the *p*-nitrophenol at the rate of 0.99, 1.81, 0.83 and 0.93 nmole min^−1^ ml^−1^, respectively (Supplementary Table S1).

## Discussion

Several literatures have reported the low bacterial diversity within the *Nepenthes* pitcher fluid^3^,^10^. The low bacterial diversity is often associated with the low pH of the *Nepenthes* juice and the presence of antibacterial and antifungal compounds in the *Nepenthes* digestive fluid which created an unfavourable environment for bacteria’s growth^2^,^3^,^5^. It is noteworthy that most studies conducted on *Nepenthes* were based on the cultured bacteria, therefore the actual bacterial diversity is yet to be discovered especially the uncultured bacteria^3^,^11^. Contrary to this, the diverse microbial distribution observed in this study suggested that conventional culture-dependent methods have indeed undermined the microbial diversity in the pitcher fluids^12^,^13^,^14^. In addition to this, 27% of the bacteria from *Nepenthes* pitcher fluid sample H1 were unclassified, as these sequences do not match to any of the sequences in the database. This suggests that the microbial diversity of *Nepenthes* pitcher fluid has a relativity higher complexity. However, the microbiota community within this quadrant remains unknown at the moment due to limitation of resources. The absence of these sequences from the database is because these bacteria were either yet to be discovered or still in the progress of characterization. Therefore, their 16S rDNA sequences are not available in the database.

Our data show that the dominant bacterial phyla in this *Nepenthes* pitcher fluid are *Proteobacteria*, *Bacteroidetes*, *Actinobacteria*, *Verrucomicrobia* and *Planctomycetes*. Former studies have reported their role in the decomposition of organic material in various environments including the gut of eukaryotes. For example, the phylum *Proteobacteria* (*Enterobactericeae*) decomposes monosaccharides and recycle nutrient of forest soil^15^. On the other hand, the phylum *Bacteroidetes* which is the major composition of human guts microbiota plays an important role in breaking down complex glycan, while the phylum *Actinobacteria* degrades starch and amylopectin in human gastrointestinal^16^,^17^. *Verrucomicrobia*, which was found in the guts of sea cucumber and termites, has been reported to be the key player in degrading complex polysaccharide^18^. The presence of these polysaccharides-degrading bacteria suggest there is a high probability that these microbes play a crucial role in the carbon recycling system within the *Nepenthes* pitcher. These microbes convert complex carbohydrates into simple sugar for the absorption of the plant via the pitcher organ. However, further study is needed in order to determine their contribution within *Nepenthes* digestive fluid.

The culture-dependent approach was conducted to confirm the presence of bacteria in the *Nepenthes* pitcher fluid. MALDI-TOF MS identification was adopted for rapid bacterial identification as it has short turn around time. Results of both identification methods, 16S rDNA and MALDI-TOF biotyping, agreed with each other (Table 1), suggests that the environmental bacterial identification by MALDI-TOF MS Biotyper is reliable. Hence, we would like to recommend the usage of MALDI-TOF MS biotyping for environmental bacterial identification owing to its fast turnover, low operating cost and simple workflow^19^,^20^.

In general, most of the *Nepenthes* live on barren soil that lacks of nitrogen source, which is an essential macronutrient for plant growth^21^. The absorption of nutrients from *Nepenthes* captive by the pitcher stimulates the nutrient uptake activity via root, and thus the later activity promotes the growth of the plant^22^. This interconnect mechanism display the importance of the pitcher for the vital plant growth^22^.

In this work, screening of the bacterial biocatalytic activities shows that a total of 12 bacterial isolates were able to degrade complex carbohydrate such as starch, xylan and cellulose. The degradation of complex polysaccharides such as tree bark, plant debris, and leaves that fallen into the *Nepenthes* pitcher fluid releases carbon as well as nitrogen into the *Nepenthes* pitcher fluid, thus nourishing the plant as well as the microbes inside the fluid^8^.

The *Nepenthes* pitcher serves as a passive trap that lures insects, which are organisms armoured by fibrous chitin and filled with protein. Therefore, the degradation of the insect cadavers involves complex proteolytic and chitinolytic activities^23^. The nitrogenous protein stored within the insect fibrous chitin and proteins embedded within the insect’s exoskeleton made up of 10% of insect’s biomass^24^,^25^,^26^. Among the 18 bacterial isolates, 9 proteases-producing and 4 chitinases-producing bacteria strains were identified as the potential contributors in insect decomposition processes. Previous study has indicated 61.5% ± 7.6% of the nitrogen within *Nepenthes* plant contributed by the decomposition of insects^27^.

Previous studies have confirmed that *Nepenthes* secretes class I, III, and IV chitinases into its digestive pitcher fluid^6^,^28^,^29^. The upregulation of the *Nepenthes* chitinase gene expression in the presence of insect or chitin and the ability to hydrolyse β-1–4 glycosidic bond of chitin has led to the suggestion that the plant chitinases plays an important role in insect degradation in *Nepenthes* pitcher^6^,^7^,^29^. The exoskeleton of insect is the main captive in *Nepenthes* pitcher which is made up of primarily chitin, a polymer of *N*-acetyl-*β*-_D_-glucosamine^30^,^31^. The chitin degradation involves the cleavage of its β-1–4 linkage from the non-reducing end by exochitinase and the random cleavage of chitin β-1–4 linkage within the chitin chain by the endochitinase^30^,^32^,^33^,^34^,^35^. The degradation of chitin involved 2 major steps, which is the cleavage of chitin into chitin oligosaccharides followed by the further cleavage to *N*-acetylglucosamine by chitobiases^31^,^36^.

In this study, 4 chitin oligosaccharides degradation bacteria were isolated from the *Nepenthes* digestive fluid. The whole genome analysis on the bacterial genome has determined 15 endo- and exochitinases. These chitinase genes were further classified into GH18 and GH19 families based on their amino acid sequences^37^,^38^. Before the documentation of GH19 family chitinase gene in *Streptomyces grisues* HUT6037, GH19 family was only found on higher plants^39^. However, at the point when this report was written, 1799 bacteria GH19 were documented in carbohydrate active enzyme (CAZY) database. Further screening has confirmed the β-*N*-acetylglucosaminidase, chitobiosidase and endochitinase activities of the chitinase genes. These suggest that the bacteria inhabit in *Nepenthes* digestive fluid cooperate with the *Nepenthes* plant for the complete digestion of the insect captive. The *Nepenthes* chitinase breakdown the chitin in the insect exoskeleton converting the chitin into chitin oligosaccharides, while the bacteria aid in the chitin oligosaccharides degradation, accelerating the nutrient recycling process.

The production of a wide range of biocatalytic enzymes by both the *Nepenthes* and bacteria inhabit in the *Nepenthes* pitcher digestive fluid contrives a dynamic environment in which both work in synchrony for the decomposition of insects, benefiting both the plant and its microbiota. This symbiotic system ensures constant essential nutrient supply from *Nepenthes* pitcher to the entire plant, thus enables the *Nepenthes* plant to survive and thrive in a nutrient-deplete environment. However, further studies need to be conducted in order to prove this hypothesis.

## Methods

### Sample Source

*Nepenthes* pitcher fluid (Sample H1) was collected from wild *Nepenthes* in Mossy Forest, Pahang, Malaysia (N 04°31′, E 101°22′), at the altitude of 1970 m above sea level. The *Nepenthes* digestive fluid was transported to laboratory and processed immediately.

### Metagenome DNA Extraction

Total genomic DNA was extracted from 35 ml of *Nepenthes* pitcher fluid by modified cetyltrimethylammonium bromide (CTAB) DNA extraction protocol. Briefly, cells were mechanically lysed with glass bead followed by chemical lysis with CTAB lysis buffer (100 mM Tris-HCl, 100 mM EDTA, 100 mM K_2_HPO_4_, 1.5 M NaCl, 1.0% (w/v) SDS and 1.0% (w/v) CTAB)^40^,^41^. Enzymatically lysis was performed by addition of lysozyme (100 μg/ml) and incubated at 37 °C for 30 min. After Proteinase K (40 μg/ml) and RNase A treatment, the DNA was purified with phenol/chloroform/isoamyl alcohol mixtures (25:24:1) and precipitated with 70.0% (v/v) ethanol. DNA pellet was rehydrated with 35 μl nuclease-free water and kept in −20 °C.

### Targeted Metagenomic Sequencing

Total DNA extracted from the *Nepenthes* fluid was subjected to 16S rDNA genes amplification with forward primer (MID1_530F, 5′-ACG AGT GCG TGT GCC AGC MGC NGC GG -3′) and reverse primer (MID1_1100modR, 5′-ACG AGT GCG TGG GTT NCG NTC GTT RC -3′)^42^. Gene amplification was preformed with gradient annealing temperature from 55 °C to 65 °C. The amplicon sequencing was performed on GS-FLX Titanium platform (Roche, USA).

### Taxonomic Assignment of Metagenomic Sequences

Sequences were trimmed with CLC genomic workbench and annotated with MG-RAST (v3.3) against RDP database^21^,^43^,^44^. Rarefaction curve and alpha diversity were constructed and calculated by comparing the data to RDP database using default setting (maximum e-value cutoff: 1e^−5^, minimum identity cutoff: 60.0% and minimum alignment cutoff: 15) in MG-RAST user interface. The bacteria abundance was calculated based on the data generated from MG-RAST.

### Culturomics Bacterial Identification

Bacteria were isolated from highland *Nepenthes* pitcher fluid (sample H1) and maintained using Luria-Bertani (LB) medium at 28 °C. The identity of the culturable bacteria was identified using MALDI-TOF MS (Bruker, Germany) equipped with Bruker FlexControl software version 3.3 and Bruker MALDI Biotyper Real Time Classification (RTC) version 3.1. This bacterial identification was performed according to the direct transfer procedure from Bruker^45^. Protein from the bacterial cell was measured by MALDI-TOF MS and the spectra generated were compared to the reference database for bacterial identification. Results with the log (score) value equal or higher than 2 indicate high confidence identification and is tabulated in Table 1.

The bacterial identities were confirmed molecularly by phylogenetic analysis on their 16S rDNA genes sequences. Bacterial genomic DNA were extracted using QIAamp DNA mini kit according to manufacturer instruction. 16S rDNA genes were amplified with 27F and 1525R primer pair^46^. Molecular identities of the isolates were identified based on the phylogenetic analysis of the 16S rDNA gene sequences using MEGA (v6.06)^47^.

### Biocatalytic Assays

Amylolytic activity of the 18 bacterial isolates was screened using diluted LB agar supplied with 0.5% (w/v) soluble starch. The utilization of starch by the bacteria was observed by flooding the starch agar with iodine. Iodine reacts with starch giving a dark blue substrate. Amylolytic bacteria degrade starch and release iodine from iodine/starch complex into the medium which results yellow halo around bacteria colonies on dark blue background.

The bacterial proteolytic activity was screened using diluted LB agar supplied with 5% (v/v) skim milk. Skim milk contributed to the opaque properties on the agar. Clear-zone around the bacterial colony will be observed on the skim milk agar for bacteria that possess proteolytic activity.

Cellulolytic activity was tested with basal salt medium supplied with 0.15% (w/v) microcrystalline cellulose, adjusted to pH 7.0. This agar was stained red with 0.02% (w/v) Congo red. The cellulose degradation by cellulolytic bacteria was identified by the Congo red discolorization which results in the formation of light yellow zone or clear zone around the colony on medium^9^.

Xylanolytic activity was screened using EnzChek^®^ ultra xylanase assay kit (Invitrogen, USA). The xylanase substrate (synthetic hemicellulose polysaccharides) was tagged with fluorescence dye and supplied by the kit. The structure of the xylanase is proprietary product to the manufacture. Xylanase by *Trichoderma viride* (Sigma, US) was used as the positive control for the xylanolytic assay. Bacterial samples were prepared by resuspending the cell pellet from 1 ml of bacteria culture into 100 μl of sterile culture medium. Working buffer (100 μl) was added to the resuspended cell before loading the bacteria cells (50 μl) into the 96 wells black plate. The assay was performed according to the protocol provided by manufacture at 25 °C. The presence of xylanase was detected by measuring the fluorescence level and compared to the standard curve. Fluorophore was released from the xylanase substrate when xylanase breaks the xylosidic lingkage of the xylanase. The fluorescence was measured at excitation/emission 360/465 using Infinite^®^ F200 PRO (Tecan, Switzerland).

Chitinase, in particular, β-*N*-acetylglucosaminidase, chitobiosidase and endochitinase activities of the bacterial isolates were tested using chitinase assay kit (Sigma, US) as described by manufacture^48^. Substrate solution was prepared by dissolving 1 mg of appropriate substrate (4-nitrophenyl *N*-acetyl-β-D-glucosaminide, 4-nitrophenyl *N*,*N*’diacetyl-β-D-chitobioside or 4-nitrophenyl β-D-*N*,*N*’,*N*”-triacetylchitotriose) in 1 ml of assay buffer. Chitinase control enzyme was diluted to 10 μg/ml with phosphate buffered saline before used. Diluted chitinase control enzyme (10 μl) was added to 90 μl of substrate solution and this reaction served as positive control in the assay. Blank reaction was prepared by loading 100 μl of substrate solution to a well in microtitre plate. Each reaction tested by adding 10 μl of overnight bacteria culture to 90 μl of substrate solution. The reaction was incubated for 24 hours followed by the addition of 200 μl of stop solution. The assay was carried out at 28 °C for bacterial culture and 37 °C for *E*. *coli* transformant carrying chitinase gene. β-*N*-acetylglucosaminidase, chitobiosidase and endochitinase producing bacteria hydrolyzed the chitinase substrate and released *p*-nitrophenol. The *p*-nitrophenol released was ionized by the stop solution (sodium carbonate) into *p*-nitrophenylate ion which was yellow color. The yellow tint measurement was taken at the absorption 405 nm using Infinite^®^ M200 (Tecan, Switzerland) and compared with the reading from chitinase control enzyme.

### Bacteria Whole Genome Sequencing

Genomic DNA of the bacterial isolates was extracted using QIAamp DNA mini kit (Qiagen, Germany) as described by the manufacturer. The quality and quantity of the DNA was checked with Nanodrop 2000c (Thermo Scientific, USA) and Qubit 2.0 fluorometer (Invitrogen, USA), respectively. Sequencing libraries were prepared with Nextera sample prep kit (Illumina, USA) and the indexing was performed according to Nextera Index kit (Illumina, USA). Prior to sequencing, the sequencing library was verified with Bioanalyzer DNA high sensitivity chip (Agilent, USA). Subsequently, 12 pM of the pooled sequencing library was loaded into MiSeq sequencing cartridge (2 × 250 bp, 500 cycles) (Illumina, USA). The sequencing was performed on MiSeq (Illumina, USA) sequencing platform.

### Bacteria Draft Genome Analysis and Gene Prediction

The quality of the sequencing raw data was checked with FastQC^49^. Subsequently, the paired-end reads were trimmed and *de novo* assembled with CLC genomic workbench version 6 (CLC, Denmark)^21^. The coding DNA sequence (CDS) was predicted using prodigal (v2.60)^50^. Draft genomes were annotated by BLASTX against NCBI non-redundant (nr) database^51^. The draft genomes were deposited on DDBJ/EMBL/GenBank.

### Chitinase Gene Analysis and Cloning

The putative chitinase genes sequences with e-value of 0.00, indicating that the sequence alignment was not occurred by chance, were preceded for further analysis^52^. The low e-value indicates that the match of the query gene against the database was significant. The chitinase genes were group by phylogenetic analysis using MEGA (v6.06) by its amino acid sequences^47^. These genes were synthesized and inserted into pET−23(+) expression vector by service provider, Genscript. Subsequently, the vector was transformed into *Escherichia coli* BL21 and preceded to chitinolytic assays.

## Additional Information

**Accession Codes**: Bacteria 16S rDNA sequences are available at DDBJ/EMBL/GenBank with the accession number KF557585- KF557587, KF557591-KF557597, KF557599-KF557602 and KF742682- KF742685. The bacteria whole genome sequences are available at DDBJ/EMBL/GenBank with the accession number AYME00000000 – AYMV00000000. The putative chitinase genes sequences were deposited in DDBJ/EMBL/GenBank and accessible with the accession numbers KT921876 - KT921890 KT921876 - KT921890.

**How to cite this article**: Chan, X.-Y. *et al.* Microbiome and Biocatalytic Bacteria in Monkey Cup (*Nepenthes* Pitcher) Digestive Fluid. *Sci. Rep.* **6**, 20016; doi: 10.1038/srep20016 (2016).

## Supplementary Material

Supplementary Information

## Figures and Tables

**Table 1:** The identity of bacteria isolated from *Nepenthes* pitcher fluid H1.

Strain	16S rDNA	MALDI-TOF MS	16S rDNA Gene Accession Number
H1a	*Bacillus* sp.	*Bacillus mycoides*	KF557587
H1g	*Klebsiella oxytoca*	*Klebsiella oxytoca*	KF557591
H1h	*Pseudomonas* sp.	*Pseudomonas koreensis*	KF557592
H1k	*Lysinibacillus fusiformis*	*Lysinibacillus fusiformis*	KF557593
H1l	*Pseudomonas aeruginosa*	*Pseudomonas aeruginosa*	KF557594
H1m	*Bacillus* sp.	*Bacillus thuringiensis*	KF557595
H1n	*Serratia fonticola*	*Serratia fonticola*	KF557596
H1q	*Serratia marcescens*	*Serratia marcescens*	KF557597
H1r	*Morganella morganii*	*Morganella morganii*	KF742682
H1w	*Serratia fonticola*	*Serratia fonticola*	KF557599
H1ai	*Sphingobacterium* sp.	Not identified	KF742683
H1aii	*Leifsonia aquatica*	Not identified	KF742684
H1bi	*Myroides odoratimimus*	*Myroides odoratimimus*	KF742685
DH1b	*Microbacterium paraoxydans*	*Microbacterium* sp.	KF557585
DH1f	*Achromobacter* sp.	*Achromobacter spanius*	KF557586
PH1a	*Serratia marcescens*	*Serratia marcescens*	KF557600
PH1b	*Pseudomonas* sp.	*Pseudomonas corrugate*	KF557601
PH1c	*Leucobacter* sp.	Not identified	KF557602

Bacteria were identified by 16S rDNA gene sequences phylogenetic analysis and MALDI-TOF MS analysis.

**Table 2:** Screening of amylolytic, proteolytic, cellulolytic, xylanolytic and chitinolytic activities from bacteria isolated from *Nepenthes* pitcher fluid H1.

Strain	Identity	Proteolytic	Amylolytic	Cellulolytic	Xylanolytic	Chitinolytic
H1a	*Bacillus* sp.	**+**	**+**	**+**	**−**	**−**
H1g	*Klebsiella oxytoca*	**−**	**−**	**−**	**−**	**+**
H1h	*Pseudomonas* sp.	**−**	**−**	**+**	**+**	**−**
H1k	*Lysinibacillus fusiformis*	**−**	**−**	**−**	**−**	**−**
H1l	*Pseudomonas aeruginosa*	**+**	**−**	**−**	**+**	**−**
H1m	*Bacillus* sp.	**+**	**+**	**+**	**−**	**−**
H1n	*Serratia fonticola*	**−**	**−**	**−**	**−**	**−**
H1q	*Serratia marcescens*	**+**	**−**	**−**	**−**	**+**
H1r	*Morganella morganii*	**−**	**−**	**−**	**−**	**−**
H1w	*Serratia* sp.	**−**	**−**	**−**	**−**	**−**
H1ai	*Sphingobacterium* sp.	**+**	**+**	**+**	**+**	**−**
H1aii	*Leifsonia aquatica*	**−**	**−**	**−**	**−**	**−**
H1bi	*Myroides odoratimimus*	**+**	**−**	**+**	**−**	**−**
DH1b	*Microbacterium paraoxydans*	**+**	**−**	**+**	**−**	**−**
DH1f	*Achromobacter* sp.	**−**	**−**	**−**	**−**	**−**
PH1a	*Serratia marcescens*	**+**	**−**	**+**	**−**	**+**
PH1b	*Pseudomonas* sp.	**+**	**−**	**+**	**+**	**+**
PH1c	*Leucobacter* sp.	**−**	**−**	**−**	**−**	**−**

+: positive result, −: negative result.

**Table 3:** Bacteria Genome Sequence Analysis.

Strain	Bacteria Identity	Draft Genome Size (Mbp)	Average Coverage	GC content (%)	Number of Contigs	GenBank Accession Number
H1a	*Bacillus* sp.	5.8	120	35.0	167	AYMH00000000
H1g	*Klebsiella oxytoca*	5.8	142	56.0	68	AYMI00000000
H1h	*Pseudomonas* sp.	6.4	74	60.3	78	AYMJ00000000
H1k	*Lysinibacillus fusiformis*	4.8	87	37.3	60	AYMK00000000
H1l	*Pseudomonas aeruginosa*	6.4	72	66.4	40	AYML00000000
H1m	*Bacillus* sp.	5.7	34	35.0	178	AYMM00000000
H1n	*Serratia fonticola*	6.1	58	53.8	247	AYMN00000000
H1q	*Serratia marcescens*	5.2	132	59.2	60	AYMO00000000
H1r	*Morganella morganii*	4.5	85	50.1	118	AYMP00000000
H1w	*Serratia fonticola*	6.1	88	53.8	240	AYMQ00000000
H1ai	*Sphingobacterium* sp.	6.7	88	39.0	66	AYMG00000000
H1aii	*Leifsonia aquatic*	4.6	108	70.4	73	AYMR00000000
H1bi	*Myroides odoratimimus*	3.9	72	34.0	183	AYMS00000000
DH1b	*Microbacterium paraoxydans*	3.6	153	70.2	47	AYME00000000
DH1f	*Achromobacter* sp.	7.1	105	66.4	148	AYMF00000000
PH1a	*Serratia marcescens*	5.2	86	59.2	56	AYMT00000000
PH1b	*Pseudomonas* sp.	7.4	94	62.9	89	AYMU00000000
PH1c	*Leucobacter* sp.	3.1	108	71.3	73	AYMV00000000

Bacteria were sequenced to the average coverage of at least 30-fold and assembled less than 250 contigs. The draft genomes sequences were deposited at DDBJ/EMBL/GenBank.

## References

[b1] Owen T. P. & Lennon K. A. Structure and development of the pitchers from the carnivorous plant *Nepenthes alata* (*Nepenthaceae*). Am. J. Bot. 86, 1382–1390 (1999). 10523280

[b2] Buch F. *et al.* Secreted pitfall-trap fluid of carnivorous *Nepenthes* plants is unsuitable for microbial growth. Ann. Bot. (Lond.) 111, 375–383 (2013). 10.1093/aob/mcs287 PMC3579442 23264234

[b3] Higashi S., Nakashima A., Ozaki H., Abe M. & Uchiumi T. Analysis of feeding mechanism in a pitcher of *Nepenthes hybrida*. J. Plant Res. 106, 47–54 (1993).

[b4] Eilenberg H. *et al.* Induced production of antifungal naphthoquinones in the pitchers of the carnivorous plant *Nepenthes khasiana*. J. Exp. Bot. 61, 911–922 (2010). 20018905 10.1093/jxb/erp359 PMC2814117

[b5] Hatano N. & Hamada T. Proteomic analysis of secreted protein induced by a component of prey in pitcher fluid of the carnivorous plant *Nepenthes alata*. J. Proteomics 75, 4844–4852 (2012). 22705321 10.1016/j.jprot.2012.05.048

[b6] Rottloff S. *et al.* Functional characterization of a class III acid endochitinase from the traps of the carnivorous pitcher plant genus, Nepenthes. J. Exp. Bot. 62, 4639–4647 (2011). 21633084 10.1093/jxb/err173 PMC3170555

[b7] Eilenberg H., Pnini-Cohen S., Schuster S., Movtchan A. & Zilberstein A. Isolation and characterization of chitinase genes from pitchers of the carnivorous plant *Nepenthes khasiana*. J. Exp. Bot. 57, 2775–2784 (2006). 16829546 10.1093/jxb/erl048

[b8] Melillo J. M., Aber J. D. & Muratore J. F. Nitrogen and lignin control of hardwood leaf litter decomposition dynamics. Ecology 63, 621–626 (1982).

[b9] Jo W.-S. *et al.* Development of detection methods for cellulolytic activity of *Auricularia auricula-judae*. *Mycobiology* 38, 74–77 (2010). 10.4489/MYCO.2010.38.1.074 PMC3741601 23956630

[b10] Morohoshi T. *et al.* Isolation and characterization of novel lipases from a metagenomic library of the microbial community in the pitcher fluid of the carnivorous plant *Nepenthes hybrida*. J.Biosci. Bioeng. 112, 315–320 (2011). 21778111 10.1016/j.jbiosc.2011.06.010

[b11] Adlassnig W., Peroutka M. & Lendl T. Traps of carnivorous pitcher plants as a habitat: composition of the fluid, biodiversity and mutualistic activities. Ann. Bot. (Lond.) 107, 181–194 (2011). 10.1093/aob/mcq238 PMC3025736 21159782

[b12] Koopman M. M. & Carstens B. C. The microbial phyllogeography of the carnivorous plant *Sarracenia alata*. Microb. Ecol. 61, 750–758 (2011). 21431933 10.1007/s00248-011-9832-9

[b13] Koopman M. M., Fuselier D. M., Hird S. & Carstens B. C. The carnivorous pale pitcher plant harbors diverse, distinct, and time-dependent bacterial communities. Appl. Environ. Microbiol. 76, 1851–1860 (2010). 20097807 10.1128/AEM.02440-09 PMC2838028

[b14] Krieger J. R. & Kourtev P. S. Bacterial diversity in three distinct sub‐habitats within the pitchers of the northern pitcher plant, Sarracenia purpurea. FEMS Microbiol. Ecol. 79, 555–567 (2012). 22092381 10.1111/j.1574-6941.2011.01240.x

[b15] Degelmann D. M., Kolb S., Dumont M., Murrell J. C. & Drake H. L. *Enterobacteriaceae* facilitate the anaerobic degradation of glucose by a forest soil. FEMS Microbiol. Ecol. 68, 312–319 (2009). 19453494 10.1111/j.1574-6941.2009.00681.x

[b16] Martens E. C., Koropatkin N. M., Smith T. J. & Gordon J. I. Complex glycan catabolism by the human gut microbiota: the *Bacteroidetes sus*-like paradigm. J. Biol. Chem. 284, 24673–24677 (2009). 19553672 10.1074/jbc.R109.022848 PMC2757170

[b17] Ryan S. M., Fitzgerald G. F. & van Sinderen D. Screening for and identification of starch-, amylopectin-, and pullulan-degrading activities in bifidobacterial strains. Appl. Environ. Microbiol. 72, 5289–5296 (2006). 16885278 10.1128/AEM.00257-06 PMC1538741

[b18] Wagner M. & Horn M. The *Planctomycetes*, *Verrucomicrobia*, *Chlamydiae* and sister phyla comprise a superphylum with biotechnological and medical relevance. Curr. Opin. Biotechnol. 17, 241–249 (2006). 16704931 10.1016/j.copbio.2006.05.005

[b19] Maier T., Klepel S., Renner U. & Kostrzewa M. Fast and reliable maldi-tof ms–based microorganism identification. Nature Methods Application Notes 3 (2006). 10.1038/nmeth870.

[b20] Ferreira L. *et al.* Identification of *Brucella* by MALDI-TOF mass spectrometry. Fast and reliable identification from agar plates and blood cultures. PLoS One 5, e14235 (2010). 21151913 10.1371/journal.pone.0014235 PMC2997794

[b21] Chan X. Y., Chua K. H., Puthucheary S. D., Yin W.-F. & Chan K.-G. Draft genome sequence of an *Aeromonas* sp. strain 159 clinical isolate that shows quorum-sensing activity. J. Bacteriol. 194, 6350–6350 (2012). 23105081 10.1128/JB.01642-12 PMC3486352

[b22] Adamec L. Foliar mineral nutrient uptake in carnivorous plants: what do we know and what should we know? Front. Plant Sci. 4, 1–3 (2013). 10.3389/fpls.2013.00010. 23386858 PMC3560283

[b23] Kato M., Hotta M., Tamin R. & Itino T. Inter-and intra-specific variation in prey assemblages and inhabitant communities in *Nepenthes* pitchers in Sumatra. Trop. Zool. 6, 11–25 (1993).

[b24] Juniper B. B. E., Robins R. J. & Joel D. M. The Carnivorous Plants. (Academic Press, 1989).

[b25] Vincent J. F. & Wegst U. G. Design and mechanical properties of insect cuticle. Arthropod Struct. Dev. 33, 187–199 (2004). 18089034 10.1016/j.asd.2004.05.006

[b26] Fagan W. F. *et al.* Nitrogen in insects: implications for trophic complexity and species diversification. Am. Nat. 160, 784–802 (2002). 18707465 10.1086/343879

[b27] Schulze W., Schulze E., Pate J. & Gillison A. The nitrogen supply from soils and insects during growth of the pitcher plants *Nepenthes mirabilis*, *Cephalotus follicularis* and *Darlingtonia californica*. Oecologia 112, 464–471 (1997). 10.1007/s004420050333 28307622

[b28] Renner T. & Specht C. D. Molecular and functional evolution of class I chitinases for plant carnivory in the *Caryophyllales*. Mol. Biol. Evol. 29, 2971–2985 (2012). 22490823 10.1093/molbev/mss106

[b29] Ishisaki K., Honda Y., Taniguchi H., Hatano N. & Hamada T. Heterogonous expression and characterization of a plant class IV chitinase from the pitcher of the carnivorous plant *Nepenthes alata*. Glycobiology 22, 345–351 (2012). 21930651 10.1093/glycob/cwr142

[b30] Funkhouser J. D. & Aronson N. N. Chitinase family GH18: evolutionary insights from the genomic history of a diverse protein family. BMC Evol. Biol. 7, 96 (2007). 17594485 10.1186/1471-2148-7-96 PMC1945033

[b31] Hamid R. *et al.* Chitinases: an update. J. Pharm. Bioall. Sci. 5, 21 (2013). 10.4103/0975-7406.106559 PMC3612335 23559820

[b32] Huang Q.-S. *et al.* The GH18 family of chitinases: their domain architectures, functions and evolutions. Glycobiology 22, 23–34 (2012). 21750098 10.1093/glycob/cwr092

[b33] Van Aalten D. *et al.* Structural insights into the catalytic mechanism of a family 18 exo-chitinase. Proc. Natl. Acad. Sci. 98, 8979–8984 (2001). 11481469 10.1073/pnas.151103798 PMC55359

[b34] Tronsmo A. & Harman G. E. Detection and Quantification of *N*-Acetyl-β-D-glucosaminidase, chitobiosidase, and endochitinase in solutions and on gels. Analytical Biochemistry 208, 74–79 (1993). 8434798 10.1006/abio.1993.1010

[b35] Brurberg M. B., Nes I. F. & Eijsink V. G. Comparative studies of chitinases A and B from *Serratia marcescens*. Microbiology 142, 1581–1589 (1996). 8757722 10.1099/13500872-142-7-1581

[b36] Suginta W., Robertson P., Austin B., Fry S. C. & Fothergill‐Gilmore L. A. Chitinases from *Vibrio*: activity screening and purification of chiA from *Vibrio carchariae*. J. Appl. Microbiol. 89, 76–84 (2000). 10945782 10.1046/j.1365-2672.2000.01076.x

[b37] Ohnuma T. *et al.* Chitin oligosaccharide binding to a family GH19 chitinase from the moss *Bryum coronatum*. FEBS J. 278, 3991–4001 (2011). 21838762 10.1111/j.1742-4658.2011.08301.x

[b38] Kawase T. *et al.* Comparison of enzymatic and antifungal properties between family 18 and 19 chitinases from *S. coelicolor* A3 (2). Biosci. Biotechnol. Biochem. 70, 988–998 (2006). 16636468 10.1271/bbb.70.988

[b39] Ohno T. *et al.* A modular family 19 chitinase found in the prokaryotic organism *Streptomyces griseus* HUT 6037. J. Bacteriol. 178, 5065–5070 (1996). 8752320 10.1128/jb.178.17.5065-5070.1996 PMC178299

[b40] Murray M. & Thompson W. F. Rapid isolation of high molecular weight plant DNA. Nucleic Acids Res. 8, 4321–4326 (1980). 7433111 10.1093/nar/8.19.4321 PMC324241

[b41] Zhou J., Bruns M. A. & Tiedje J. M. DNA recovery from soils of diverse composition. Appl. Environ. Microbiol. 62, 316–322 (1996). 8593035 10.1128/aem.62.2.316-322.1996 PMC167800

[b42] Wang Y. & Qian P.-Y. Conservative fragments in bacterial 16S rRNA genes and primer design for 16S ribosomal DNA amplicons in metagenomic studies. PloS One 4, e7401 (2009). 19816594 10.1371/journal.pone.0007401 PMC2754607

[b43] Meyer F. *et al.* The metagenomics RAST server–a public resource for the automatic phylogenetic and functional analysis of metagenomes. BMC Bioinformatics 9, 386 (2008). 18803844 10.1186/1471-2105-9-386 PMC2563014

[b44] Cole J. R. *et al.* The Ribosomal Database Project: improved alignments and new tools for rRNA analysis. Nucleic Acids Research 37, D141–D145 (2009). 19004872 10.1093/nar/gkn879 PMC2686447

[b45] Chen J. W., Koh C.-L., Sam C.-K., Yin W.-F. & Chan K.-G. Short chain *N*-acyl homoserine lactone production by soil isolate *Burkholderia* sp. strain A9. Sensors 13, 13217–13227 (2013). 24084115 10.3390/s131013217 PMC3859060

[b46] Chong T. M. *et al.* Characterization of quorum sensing and quorum quenching soil bacteria isolated from Malaysian tropical montane forest. Sensors 12, 4846–4859, 10.3390/s120404846 (2012). 22666062 PMC3355444

[b47] Tamura K., Stecher G., Peterson D., Filipski A. & Kumar S. MEGA6: Molecular Evolutionary Genetics Analysis Version 6.0. Mol. Biol. Evol. 30, 2725–2729 (2013). 24132122 10.1093/molbev/mst197 PMC3840312

[b48] Hurst M. R. *et al.* The main virulence determinant of *Yersinia entomophaga* MH96 is a broad-host-range toxin complex active against insects. J. Bacteriol. 193, 1966–1980 (2011). 21278295 10.1128/JB.01044-10 PMC3133040

[b49] Chan X. Y. *et al.* Insights of biosurfactant producing *Serratia marcescens* strain W2. 3 isolated from diseased tilapia fish: a draft genome analysis. *Gut* Pathogens 5, 29 (2013). 10.1186/1757-4749-5-29 PMC3816309 24148830

[b50] Doug H. *et al.* Prodigal: prokaryotic gene recognition and translation initiation site identification. BMC Bioinformatics 11, 119–130. 10.1186/1471-2105-11-119 (2010). 20211023 PMC2848648

[b51] Acland A. *et al.* Database resources of the national center for biotechnology information. Nucleic Acids Research 41, D8–D20 (2013). 23193264 10.1093/nar/gks1189 PMC3531099

[b52] Fassler J. & Cooper P. BLAST Glossary. (2011). Available at : http://www.ncbi.nlm.nih.gov/books/NBK62051/ (Accessed: 22nd November 2015).

