# Supplementary material for: Microbiome and Biocatalytic Bacteria in Monkey Cup (Nepenthes Pitcher) Digestive Fluid
Source: Sci Rep. 2016 Jan 28;6:20016. doi: 10.1038/srep20016 (PMC4730220; doi:10.1038/srep20016)
Supplement: Supplementary Information [file srep20016-s1.pdf]

Title: Microbiome and Biocatalytic Bacteria in Monkey Cup (*Nepenthes* Pitcher) Digestive Fluid

Xin-Yue Chan[1], Kar-Wai Hong[1], Wai-Fong Yin[1], and Kok-Gan Chan[1*]

[1] Division of Genetics and Molecular Biology, Institute of Biological Sciences, Faculty of Science, University of Malaya, 50603 Malaysia

Corresponding Author; Email: kokgan@um.edu.my; Tel.: +603-7967-5162; Fax: +603-7967-4509

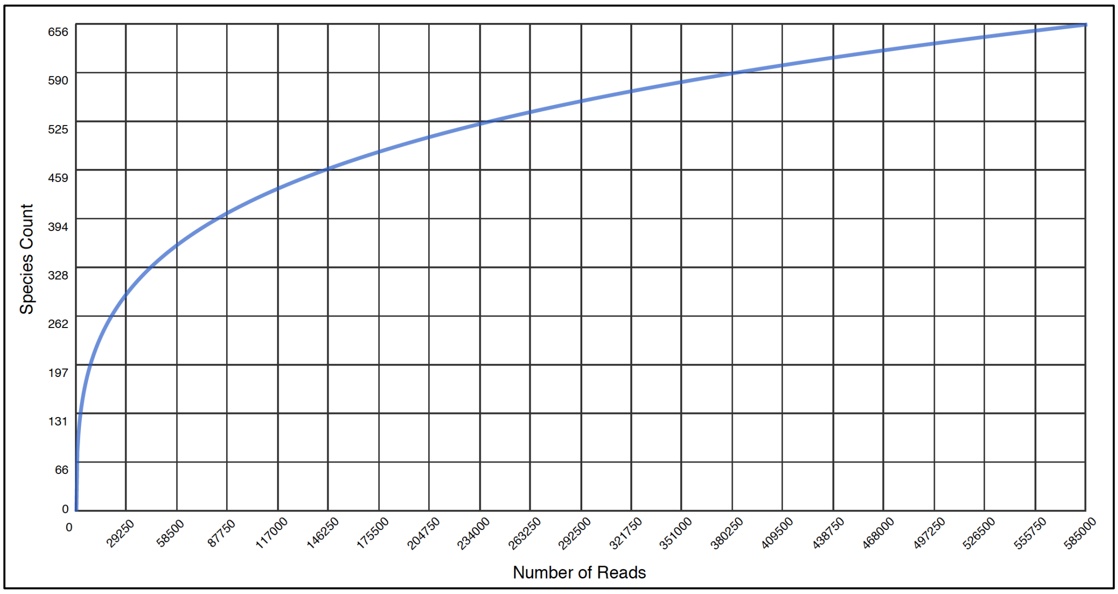

Supplementary Figure S1: Rarefaction curve of *Nepenthes* pitcher fluid sample H1. The rarefaction curve was plotted with cumulative number of individuals. The alpha diversity is 31.44.

Supplementary Table S1. β-*N*-acetylglucosaminidase, chitobiosidase and endochitinase activities from bacterial isolates. These enzymes hydrolysed nonreducing *N*-acetyl-*β*-glucosaminidase from terminal non reducing end of the chitinase substrate, releasing *p*-nitrophenol from substrate 4-nitrophenyl *N*-acetyl-*β*-D-glucosaminide, 4-nitrophenyl *N,N*'diacetyl-*β*-D-chitobioside and 4-nitrophenyl *β*- D-*N,N*',N"-triacetylchitotriose.

Sample	Bacteria Identity	β-*N*-acetylglucosaminidase (nmole min^{-1} ml^{-1})	Chitobiosidase (nmole min^{-1} ml^{-1})	Endochitinase (nmole min^{-1} ml^{-1})
		4-nitrophenyl *N*-acetyl-*β*-D-glucosaminide	4-nitrophenyl *N,N*'diacetyl-*β*-D-chitobioside	4-nitrophenyl *β*- D-*N,N*',N"-triacetylchitotriose
**Bacteria Strain**				
**Blank (Substrate only)**	N/A	0.00	0.00	0.00
**Chitinase**	N/A	73.70	71.93	58.90
**H1a**	*Bacillus* sp.	0.07	0.21	0.03
**H1g**	*Klebsiella oxytoca*	0.26	**2.38**	0.00
**H1h**	*Pseudomonas* sp.	0.12	0.14	0.05
**H1k**	*Lysinibacillus fusiformis*	0.07	0.23	0.02
**H1l**	*Pseudomonas aeruginosa*	0.08	0.18	0.06
**H1m**	*Bacillus* sp.	0.08	0.19	0.03
**H1n**	*Serratia fonticola*	0.17	0.30	0.05
**H1q**	*Serratia marcescens*	**2.15**	**3.75**	**2.54**
**H1r**	*Morganella morganii*	0.08	0.16	0.05
**H1w**	*Serratia fonticola*	0.20	0.24	0.16
**H1ai**	*Sphingobacterium* sp.	0.08	0.38	0.04
**H1aii**	*Leifsonia aquatic*	0.05	0.16	0.07

**H1bi**	*Myroides odoratimimus*	0.08	0.21	0.03
**DH1b**	*Microbacterium paraoxydans*	0.03	0.22	0.04
**DH1f**	*Achromobacter* sp.	0.03	0.17	0.09
**PH1a**	*Serratia marcescens*	**2.15**	**3.86**	**2.06**
**PH1b**	*Pseudomonas* sp.	0.02	**3.79**	**1.41**
**PH1c**	*Leucobacter* sp.	0.10	0.14	0.00
**Transformant**				
**Blank (Substrate only)**	N/A	0.00	0.00	0.00
**Chitinase**	N/A	77.60	93.55	76.71
**BL21**	*Escherichia coli*	0.16	0.12	0.13
**H1g contig 15**	N/A	**1.58**	0.14	0.13
**H1g contig 18**	N/A	0.17	0.11	0.13
**H1h contig 3**	N/A	0.17	**3.70**	**0.99**
**H1h contig 30**	N/A	0.17	0.12	0.13
**H1l contig 14**	N/A	0.19	**2.47**	**1.81**
**H1m contig 141**	N/A	0.19	**4.56**	**0.83**
**H1m contig 5**	N/A	0.18	0.13	0.15
**H1n contig 36**	N/A	**1.45**	0.14	0.13
**H1q contig 3**	N/A	0.18	**1.97**	**0.93**
**H1q contig 12**	N/A	0.00	0.24	0.13
**H1q contig 9**	N/A	0.15	0.11	0.10

Supplementary Table S2. Putative chitinase genes and the GenBank Accession Number. Putative chitinase gene sequences were extracted from the bacteria genomes and deposited in DDBJ/EMBL/GenBank.

Putative Chitinase Genes	GenBank Accession Number
**H1g contig 15**	KT921876
**H1g contig 18**	KT921877
**H1h contig 3**	KT921878
**H1h contig 30**	KT921879
**H1l contig 14**	KT921880
**H1m contig 5**	KT921881
**H1m contig 141**	KT921882
**H1n contig 36**	KT921883
**H1q contig 3**	KT921884
**H1q contig 9**	KT921885
**H1q contig 12**	KT921886
**H1w contig 37**	KT921887
**PH1a contig 10**	KT921888
**PH1a contig 12**	KT921889
**PH1a contig 30**	KT921890
